# High temporal-resolution phase-contrast MRI demonstrates impaired left-ventricular diastolic relaxation in mice fed a high-fat high-sucrose diet

**DOI:** 10.1186/1532-429X-18-S1-P50

**Published:** 2016-01-27

**Authors:** Nivedita K Naresh, Yang Yang, Jeffrey W Holmes, Michael Salerno, Frederick H Epstein

**Affiliations:** 1 grid.27755.32000000009136933XBiomedical Engineering, University of Virginia, Charlottesville, VA USA; 2 grid.27755.32000000009136933XMedicine, University of Virginia, Charlottesville, VA USA; 3 grid.27755.32000000009136933XRadiology, University of Virginia, Charlottesville, VA USA

## Background

More than one-third of U.S. adults are obese, incurring an increased risk of heart failure^1^. Increased body mass index is associated with left-ventricular (LV) diastolic dysfunction, which may be one of the pathophysiological links between obesity and heart failure^2^. Mouse models can elucidate molecular mechanisms that underlie cardiovascular disease. In this study we used high temporal-resolution phase-contrast MRI to test the hypothesis that mice fed a high-fat high-sucrose diet (HFHSD) have diastolic dysfunction.

## Methods

C57Bl/6 mice fed a regular chow diet (n = 5) or a HFHSD (Research Diets D12327, n = 8) for 22 weeks were imaged on a 7T system. Mice were anesthetized with 1.25% isoflurane and maintained at 36 ± 1°C during MRI. During imaging, physiological monitoring and gating of the ECG and respiration were performed using an MRI-compatible system (SAII, Stony Brook, NY). Localizer and long-axis imaging were performed to select an imaging plane containing both the mitral and aortic valves. High temporal-resolution 2D spiral phase-contrast MRI was performed to assess diastolic function. Imaging parameters included: TE/TR = 0.91-0.93 ms/2.5-3.3 ms, slice thickness = 1-1.25 mm, flip angle=20^0^, number of spiral interleaves = 198-350, field of view = 25 × 25 mm^2^, maximum velocity encoding = 100 cm/s in the through-plane direction, acquisition window = 200 ms and scan time=1.2 min. For all mice, the trans-mitral and trans-aortic velocity-time curves were used to quantify the maximal E and A wave velocities, E/A ratio, deceleration time (DT), ejection time (ET), isovolumetric relaxation time (IVRT) and isovolumetric contraction time (IVCT). The Tei index, which is an index of LV stiffness was also estimated as (IVRT+IVCT)/ET. The MRI protocol also included cine and DENSE MRI to quantify ejection fraction (EF) and peak circumferential strain (E_cc_).

## Results

**Figure 1:**
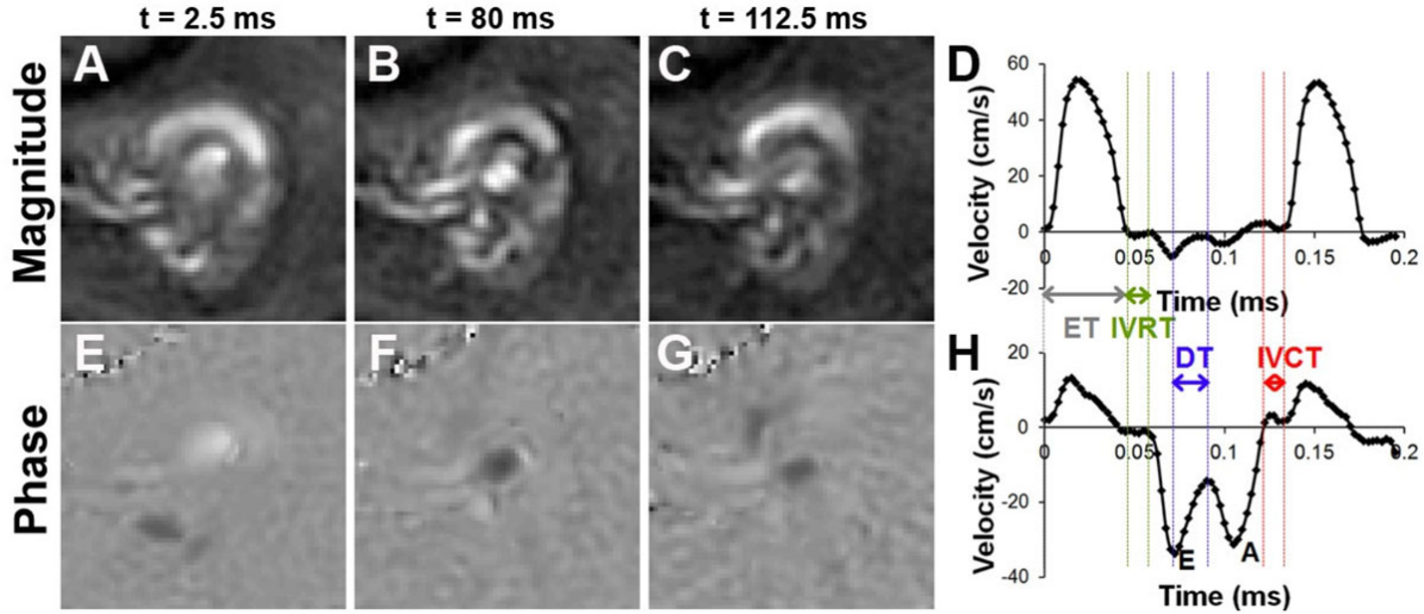
**Example magnitude (A-C) and phase images (E-G) obtained using the spiral phase contrast sequence demonstrate the trans-aortic velocities obtained at peak systole (A,E) and the trans-mitral velocities obtained at early diastole (B,F) and late diastole (C,G) in a mouse heart**. Example trans-aortic velocity-time curve (D) and trans-mitral velocity-time curve (H) obtained in a mouse using phase contrast MRI. Using the trans-mitral velocity-time curve, the early (E) and late (A) ventricular filling velocities and deceleration time (DT) can be estimated. Using the trans-aortic and trans-mitral velocity-time curve, the ejection time (ET) can be estimated as the time between the opening and closure of aortic valve. Isovolumetric relaxation time (IVRT) is estimated as the time between the closure of the aortic valve and the opening of the mitral valve. Isovolumetric contraction time (IVCT) is estimated as the time between the closure of the mitral valve and the opening of the aortic valve.

Figure 1 shows example magnitude (A-C) and phase (E-G) images obtained using phase-contrast MRI. Figure 1 also shows example trans-aortic (D) and trans-mitral (H) velocity-time curves and the definitions of the various parameters estimated from these curves. Table 1 summarizes the various parameters that were estimated for the two groups of mice. Body weight was increased by 33% (p < 0.05 vs. Control) and IVRT was increased by 50% in the HFHSD mice (p < 0.05 vs. Control). No significant differences were detected between the remaining parameters. EF and E_cc_ were normal in HFHSD mice.

**Table 1:** Parameters estimated for the control and HFHSD mice

	Control	HFHSD
**Body weight (g)**	30 ± 4	40 ± 4*
**E wave velocity (cm/s)**	37 ± 3	43 ± 7
**A wave velocity (cm/s)**	34 ± 4	34 ± 5
**E/A**	1.08 ± 0.08	1.28 ± 0.25
**DT (ms)**	16 ± 3	19 ± 7
**IVRT (ms)**	10 ± 2	15 ± 5*
**ET (ms)**	49 ± 4	51 ± 5
**IVCT (ms)**	10 ± 3	7 ± 2
**(IVRT+IVCT)/ET**	0.40 ± 0.12	0.45 ± 0.09
**R-R (ms)**	132 ± 6	136 ± 16

*p < 0.05 vs. Control

## Conclusions

High temporal-resolution phase-contrast MRI can be used to assess diastolic function in mice, with a total scan time of 1.2 minutes. The finding of increased IVRT but normal E/A ratio after 22 weeks on diet suggests that the HFHSD mice have impaired diastolic relaxation, but do not have impaired LV compliance. Future studies using cardiac MR in gene-modified mice fed a HFHS diet may shed light on molecular mechanisms underlying diastolic dysfunction in obesity related cardiomyopathy.

